# Crystal structure of 2-meth­oxy-2-[(4-methyl­phen­yl)sulfan­yl]-1-phenyl­ethan-1-one

**DOI:** 10.1107/S205698901402550X

**Published:** 2015-01-01

**Authors:** Julio Zukerman-Schpector, Paulo R. Olivato, Henrique J. Traesel, Jéssica Valença, Daniel N. S. Rodrigues, Edward R. T. Tiekink

**Affiliations:** a Departmento de Química, Universidade Federal de São Carlos, 13565-905 São Carlos, SP, Brazil; b Instituto de Química, Universidade de São Paulo, 05508-000 São Paulo, SP, Brazil; c Department of Chemistry, University of Malaya, 50603 Kuala Lumpur, Malaysia

**Keywords:** crystal structure, β-thio­carbon­yl, C—H⋯O inter­actions

## Abstract

In the title β-thio­carbonyl compound, C_16_H_16_O_2_S, the carbonyl and meth­oxy O atoms are approximately coplanar [O—C—C—O torsion angle = −18.2 (5)°] and *syn* to each other, and the tolyl ring is orientated to lie over them. The dihedral angle between the planes of the two rings is 44.03 (16)°. In the crystal, supra­molecular chains are formed along the *c* axis mediated by C—H⋯O inter­actions involving methine and methyl H atoms as donors, with the carbonyl O atom accepting both bonds; these pack with no specific inter­molecular inter­actions between them.

## Related literature   

For general background to β-thio­carbonyl and β-bis­(thio­carbon­yl) compounds, see: Vinhato *et al.* (2013 ▸); Zukerman-Schpector *et al.* (2008 ▸). For related structures, see: Olivato *et al.* (2013 ▸); Distefano *et al.* (1996 ▸). For further synthetic details, see: Ali & McDermott (2002 ▸); Zoretic & Soja (1976 ▸).
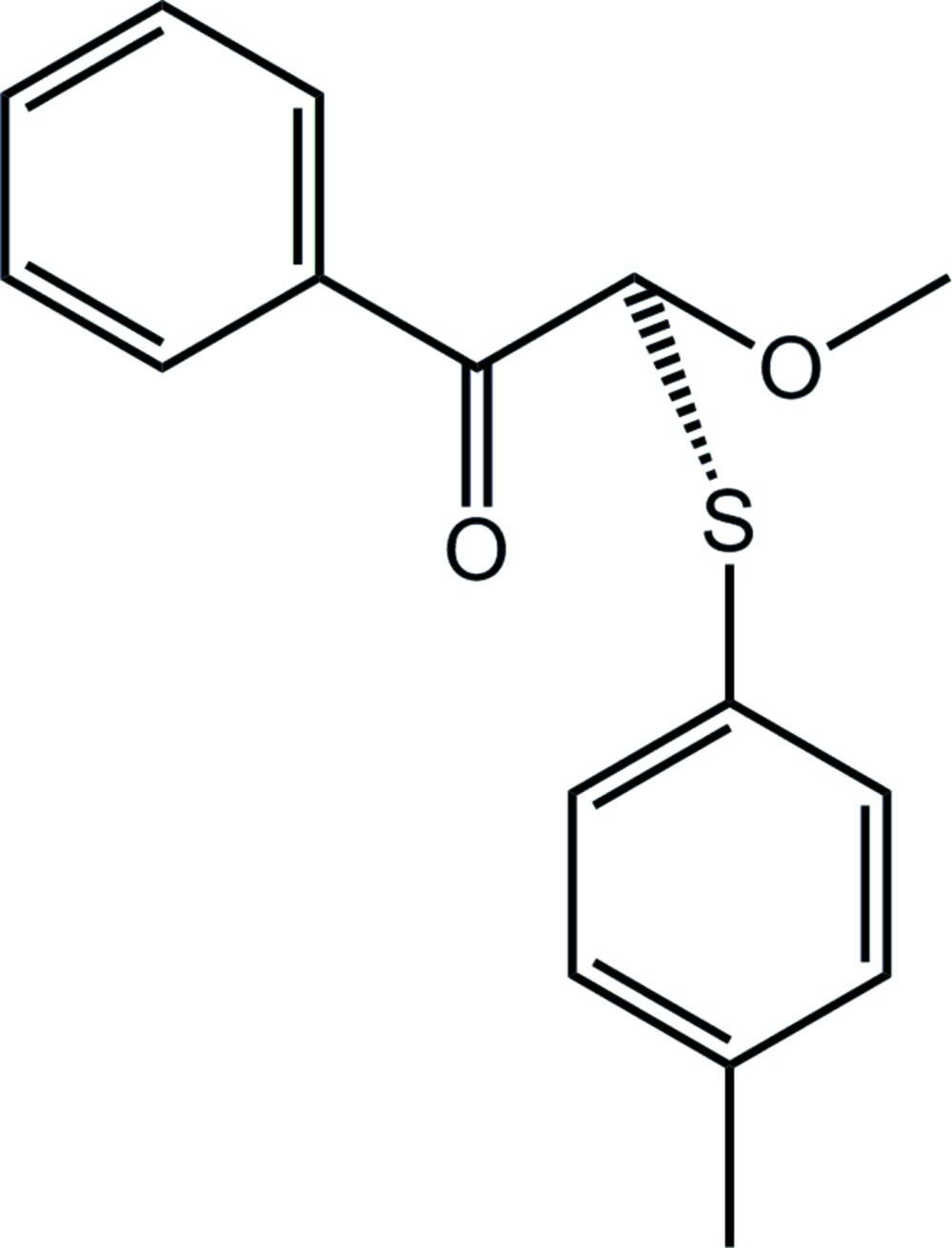


## Experimental   

### Crystal data  

C_16_H_16_O_2_S
*M* *_r_* = 272.35
Orthorhombic, 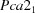

*a* = 17.8579 (9) Å
*b* = 8.1257 (4) Å
*c* = 9.8317 (5) Å
*V* = 1426.66 (12) Å^3^
*Z* = 4
Mo *K*α radiation
μ = 0.22 mm^−1^
*T* = 293 K
0.41 × 0.14 × 0.08 mm

### Data collection  

Bruker APEXII CCD diffractometer
Absorption correction: multi-scan (*SADABS*; Sheldrick, 1996 ▸) *T*_min_ = 0.690, *T*_max_ = 0.745
5399 measured reflections
2337 independent reflections
1648 reflections with *I* > 2σ(*I*)
*R*_int_ = 0.031

### Refinement  

*R*[*F* ^2^ > 2σ(*F* ^2^)] = 0.042
*wR*(*F* ^2^) = 0.090
*S* = 1.02
2337 reflections
174 parameters
1 restraint
H-atom parameters constrained
Δρ_max_ = 0.14 e Å^−3^
Δρ_min_ = −0.15 e Å^−3^
Absolute structure: Flack *x* determined using 552 quotients [(*I* ^+^)−(*I* ^−^)]/[(*I* ^+^)+(*I* ^−^)] (Parsons *et al.*, 2013 ▸)
Absolute structure parameter: 0.02 (6)

### 

Data collection: *APEX2* (Bruker, 2009 ▸); cell refinement: *SAINT* (Bruker, 2009 ▸); data reduction: *SAINT*; program(s) used to solve structure: *SIR* (Burla *et al.*, 2014 ▸; program(s) used to refine structure: *SHELXL2014* (Sheldrick, 2008 ▸); molecular graphics: *ORTEP-3 for Windows* (Farrugia, 2012 ▸) and *DIAMOND* (Brandenburg, 2006 ▸); software used to prepare material for publication: *MarvinSketch* (ChemAxon, 2010 ▸) and *publCIF* (Westrip, 2010 ▸).

## Supplementary Material

Crystal structure: contains datablock(s) I, New_Global_Publ_Block. DOI: 10.1107/S205698901402550X/hg5421sup1.cif

Structure factors: contains datablock(s) I. DOI: 10.1107/S205698901402550X/hg5421Isup2.hkl

Supporting information file. DOI: 10.1107/S205698901402550X/hg5421Isup3.cml

**Fig. 1.**
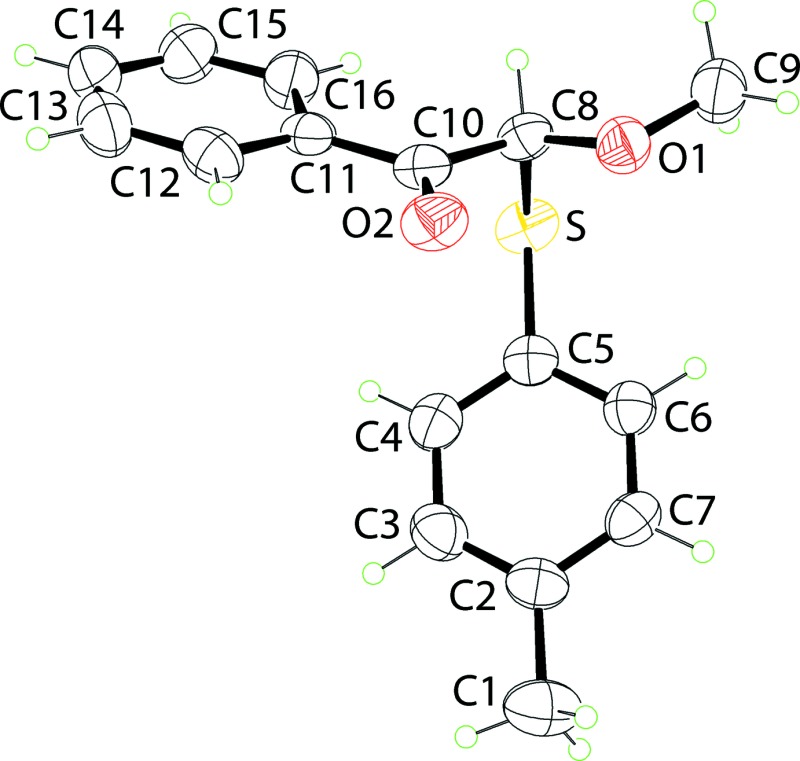
The molecular structure of the title compound showing the atom-labelling scheme and displacement ellipsoids at the 35% probability level.

**Fig. 2.**
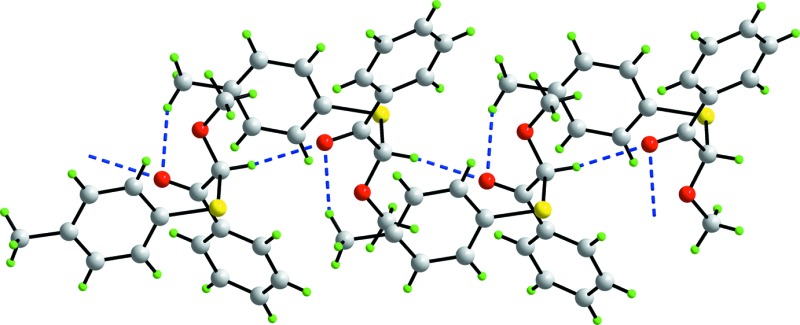
A view of the supramolecular chain along the c axis mediated by C—H···O interactions (bluee dashed lines).

**Fig. 3.**
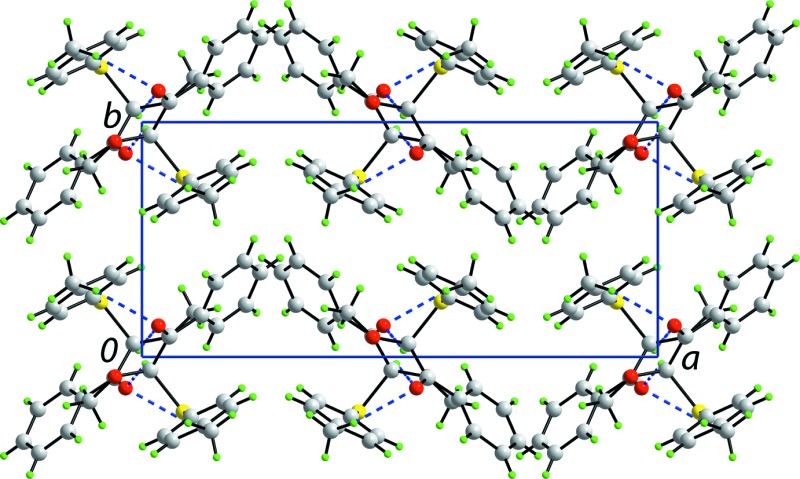
A view in projection down the c axis of the unit-cell contents. The C—H···O interactions are shown as blue dashed lines.

. DOI: 10.1107/S205698901402550X/hg5421fig1.tif
The mol­ecular structure of the title compound showing the atom-labelling scheme and displacement ellipsoids at the 35% probability level.

c . DOI: 10.1107/S205698901402550X/hg5421fig2.tif
A view of the supra­molecular chain along the *c* axis mediated by C—H⋯O inter­actions (bluee dashed lines).

c . DOI: 10.1107/S205698901402550X/hg5421fig3.tif
A view in projection down the *c* axis of the unit-cell contents. The C—H⋯O inter­actions are shown as blue dashed lines.

CCDC reference: 1035425

Additional supporting information:  crystallographic information; 3D view; checkCIF report

## Figures and Tables

**Table 1:** Hydrogen-bond geometry (, )

*D*H*A*	*D*H	H*A*	*D* *A*	*D*H*A*
C1H1*B*O2^i^	0.96	2.49	3.366(6)	152
C8H8O2^ii^	0.98	2.46	3.323(6)	146

Symmetry codes: (i) 
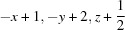
; (ii) 
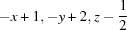
.

## supplementary crystallographic information

### S1. Introduction

As part of our on-going research on the conformational and electronic inter­actions of some β-thio-carbonyl and β-bis-thio-carbonyl compounds, e.g. N,N-di­ethyl-2-[(4-substituted) phenyl­thio]­acetamides, 1-methyl-3-phenyl­sulfonyl-2-piperidone, 3,3-bis­[(4-substituted)phenyl­sulfanyl]-1-methyl-2-piperidones, 2-alkyl­thio-2-alkyl­sulfinyl-aceto­phenones, 2-alkyl­thio-2-phenyl­sulfonyl-aceto­phenones and 2-alkyl­sulfinyl-2-alkyl­sulfonyl-aceto­phenones, utilizing spectroscopic , theoretical and X-ray diffraction methods (Vinhato *et al.*, 2013; Zukerman-Schpector *et al.*, 2008; Olivato *et al.*, 2013; Distefano *et al.*, 1996) the title compound was synthesized and its crystal structure determined.

### S2.1. Synthesis and crystallization

4-Methyl­thio­penol (5.0 g, 40 mmol) was reacted with bromine (1.1 ml, 20 mmol) in di­chloro­methane (250 mL) on hydrated silica gel support (25 g of SiO_2_ and 12 mL of water) to give 4-methyl­phenyl di­sulfide (4.1 g, yield = 83%). A white solid was obtained after filtration and evaporation without further purification (Ali & McDermott, 2002). A solution of 2-meth­oxy aceto­phenone (0.4 mL, 2.76 mmol, Sigma-Aldrich) in THF (10 ml) was added drop wise to a cooled (195 K) solution of diiso­propyl­amine (0.42 ml, 3.04 mmol) and butyl­lithium (2.0 ml, 2.76 mmol) in THF (10 ml). After 30 minutes, a solution of 4-methyl­phenyl di­sulfide (0.748 g, 3.04 mmol) with hexa­methyl­phospho­ramide (HMPA) (0.5 ml, 2.76 mmol) dissolved in THF (10 ml) was added drop wise to the enolate solution (Zoretic & Soja, 1976). After stirring for 3 h, water (50 ml) was added at room temperature and extraction with di­ethyl ether was performed. The organic layer was then treated with a saturated solution of ammonium chloride until neutral pH and dried over anhydrous magnesium sulfate. A brown oil was obtained after evaporation of the solvent. Purification through flash chromatography with n-hexane was used to remove the non-polar rea­ctant (di­sulfide) then acetone to give a mixture of both aceto­phenones (product and rea­ctant). Crystallization was performed by vapour diffusion of n-hexane into a chloro­form solution held at 283 K to give pure product (0.3 g, yield = 40%). Suitable crystals for X-ray diffraction were obtained by same pathway; m.p. 359.3–359.8 K.

^1^H NMR (CDCl_3_, 500 MHz, ppm): δ 2.33 (s, 3*H*), 3.67 (s, 3*H*), 5.81 (s, 1*H*), 7.08–7.10 (m ,2*H*), 7.23-7.25 (m, 2*H*), 7.43–7.46(m, 2*H*),7.56–7.59 (m, 1*H*), 7.95–7.96 (m, 2*H*). HRMS: calcd. for C_16_H_16_O_2_S [M + H]^+^ 272.0871; found: 272.0864.

### S2.2. Refinement

Carbon-bound H-atoms were placed in calculated positions (C—H = 0.93 to 0.98 Å) and were included in the refinement in the riding model approximation, with *U_iso_*(H) = 1.2–1.5*U_eq_*(C).

### Crystal data

**Table:**

C_16_H_16_O_2_S	*D*_x_ = 1.268 Mg m^−^^3^
*M**_r_* = 272.35	Mo *K*α radiation, λ = 0.71073 Å
Orthorhombic, *P**c**a*2_1_	Cell parameters from 1023 reflections
*a* = 17.8579 (9) Å	θ = 3.1–18.7°
*b* = 8.1257 (4) Å	µ = 0.22 mm^−^^1^
*c* = 9.8317 (5) Å	*T* = 293 K
*V* = 1426.66 (12) Å^3^	Irregular, colourless
*Z* = 4	0.41 × 0.14 × 0.08 mm
*F*(000) = 576	

### Data collection

**Table:**

Bruker APEXII CCD diffractometer	1648 reflections with *I* > 2σ(*I*)
φ and ω scans	*R*_int_ = 0.031
Absorption correction: multi-scan (*SADABS*; Sheldrick, 1996)	θ_max_ = 25.4°, θ_min_ = 2.8°
*T*_min_ = 0.690, *T*_max_ = 0.745	*h* = −21→21
5399 measured reflections	*k* = −9→9
2337 independent reflections	*l* = −10→11

### Refinement

**Table:**

Refinement on *F*^2^	Hydrogen site location: inferred from neighbouring sites
Least-squares matrix: full	H-atom parameters constrained
*R*[*F*^2^ > 2σ(*F*^2^)] = 0.042	*w* = 1/[σ^2^(*F*_o_^2^) + (0.0294*P*)^2^ + 0.2164*P*] where *P* = (*F*_o_^2^ + 2*F*_c_^2^)/3
*wR*(*F*^2^) = 0.090	(Δ/σ)_max_ < 0.001
*S* = 1.02	Δρ_max_ = 0.14 e Å^−^^3^
2337 reflections	Δρ_min_ = −0.15 e Å^−^^3^
174 parameters	Absolute structure: Flack *x* determined using 552 quotients [(*I*^+^)-(*I*^-^)]/[(*I*^+^)+(*I*^-^)] (Parsons *et al.*, 2013)
1 restraint	Absolute structure parameter: 0.02 (6)
Primary atom site location: structure-invariant direct methods	

### Special details

**Table:**

Geometry. All e.s.d.'s (except the e.s.d. in the dihedral angle between two l.s. planes) are estimated using the full covariance matrix. The cell e.s.d.'s are taken into account individually in the estimation of e.s.d.'s in distances, angles and torsion angles; correlations between e.s.d.'s in cell parameters are only used when they are defined by crystal symmetry. An approximate (isotropic) treatment of cell e.s.d.'s is used for estimating e.s.d.'s involving l.s. planes.

### Fractional atomic coordinates and isotropic or equivalent isotropic displacement parameters (Å^2^)

**Table:**

	*x*	*y*	*z*	*U*_iso_*/*U*_eq_	
C1	0.3662 (3)	0.6856 (7)	1.3764 (5)	0.0916 (18)	
H1A	0.3249	0.7532	1.4046	0.137*	
H1B	0.4106	0.7185	1.4243	0.137*	
H1C	0.3553	0.5725	1.3964	0.137*	
C2	0.3784 (3)	0.7055 (6)	1.2258 (5)	0.0642 (13)	
C3	0.3285 (3)	0.7876 (6)	1.1440 (6)	0.0737 (14)	
H3	0.2850	0.8300	1.1823	0.088*	
C4	0.3411 (3)	0.8094 (6)	1.0061 (5)	0.0683 (13)	
H4	0.3066	0.8670	0.9537	0.082*	
C5	0.4049 (2)	0.7455 (5)	0.9462 (5)	0.0564 (11)	
C6	0.4553 (2)	0.6622 (5)	1.0273 (5)	0.0615 (12)	
H6	0.4987	0.6186	0.9893	0.074*	
C7	0.4416 (3)	0.6434 (5)	1.1643 (5)	0.0635 (13)	
H7	0.4763	0.5868	1.2171	0.076*	
C8	0.4839 (2)	0.9437 (5)	0.7603 (5)	0.0576 (10)	
H8	0.4930	0.9708	0.6646	0.069*	
C9	0.6015 (3)	0.8116 (6)	0.7474 (6)	0.0834 (15)	
H9A	0.6139	0.8637	0.6627	0.125*	
H9B	0.5771	0.7085	0.7299	0.125*	
H9C	0.6465	0.7927	0.7987	0.125*	
C10	0.4468 (2)	1.0888 (5)	0.8275 (5)	0.0559 (11)	
C11	0.3852 (2)	1.1782 (5)	0.7587 (5)	0.0539 (10)	
C12	0.3573 (2)	1.3181 (5)	0.8205 (5)	0.0674 (13)	
H12	0.3767	1.3519	0.9037	0.081*	
C13	0.3007 (3)	1.4083 (5)	0.7595 (7)	0.0793 (14)	
H13	0.2828	1.5027	0.8021	0.095*	
C14	0.2709 (3)	1.3608 (6)	0.6381 (7)	0.0781 (14)	
H14	0.2325	1.4212	0.5983	0.094*	
C15	0.2984 (3)	1.2225 (7)	0.5754 (5)	0.0840 (16)	
H15	0.2780	1.1887	0.4930	0.101*	
C16	0.3556 (3)	1.1333 (6)	0.6325 (5)	0.0736 (13)	
H16	0.3748	1.0423	0.5868	0.088*	
O1	0.55239 (15)	0.9162 (4)	0.8235 (3)	0.0666 (9)	
O2	0.46803 (18)	1.1328 (4)	0.9400 (3)	0.0727 (9)	
S	0.41986 (7)	0.76542 (13)	0.76791 (16)	0.0711 (4)	

### Atomic displacement parameters (Å^2^)

**Table:**

	*U*^11^	*U*^22^	*U*^33^	*U*^12^	*U*^13^	*U*^23^
C1	0.090 (4)	0.115 (5)	0.070 (4)	−0.030 (3)	0.006 (3)	0.005 (3)
C2	0.065 (3)	0.064 (3)	0.064 (4)	−0.020 (2)	0.006 (3)	0.000 (3)
C3	0.061 (3)	0.077 (3)	0.084 (4)	−0.004 (3)	0.011 (3)	0.002 (3)
C4	0.064 (3)	0.064 (3)	0.077 (4)	−0.001 (2)	−0.007 (3)	0.006 (3)
C5	0.061 (3)	0.048 (2)	0.060 (3)	−0.011 (2)	−0.001 (2)	−0.003 (2)
C6	0.061 (3)	0.056 (3)	0.067 (3)	0.000 (2)	−0.001 (2)	−0.001 (3)
C7	0.066 (3)	0.058 (3)	0.066 (4)	−0.006 (2)	−0.008 (3)	0.009 (3)
C8	0.070 (3)	0.058 (2)	0.045 (2)	−0.0025 (19)	−0.003 (3)	0.003 (3)
C9	0.084 (3)	0.095 (3)	0.072 (4)	0.024 (3)	−0.003 (3)	0.000 (4)
C10	0.070 (3)	0.052 (3)	0.046 (3)	−0.009 (2)	0.007 (2)	0.002 (2)
C11	0.061 (2)	0.051 (2)	0.049 (3)	−0.0077 (18)	0.012 (3)	0.003 (3)
C12	0.066 (3)	0.065 (3)	0.071 (3)	−0.009 (2)	0.012 (3)	−0.013 (3)
C13	0.069 (3)	0.065 (3)	0.104 (4)	0.006 (2)	0.013 (4)	−0.008 (4)
C14	0.064 (3)	0.077 (3)	0.094 (4)	0.009 (3)	0.013 (3)	0.013 (3)
C15	0.085 (3)	0.102 (4)	0.065 (4)	0.014 (3)	−0.011 (3)	0.010 (3)
C16	0.090 (3)	0.078 (3)	0.053 (3)	0.017 (3)	−0.004 (3)	−0.007 (3)
O1	0.0665 (18)	0.077 (2)	0.057 (2)	0.0086 (16)	−0.0038 (16)	−0.0012 (16)
O2	0.094 (2)	0.076 (2)	0.048 (2)	−0.0017 (17)	−0.0047 (18)	−0.0078 (19)
S	0.0948 (8)	0.0600 (6)	0.0583 (7)	−0.0129 (6)	−0.0079 (8)	−0.0055 (8)

### Geometric parameters (Å, º)

**Table:**

C1—C2	1.505 (7)	C8—H8	0.9800
C1—H1A	0.9600	C9—O1	1.432 (5)
C1—H1B	0.9600	C9—H9A	0.9600
C1—H1C	0.9600	C9—H9B	0.9600
C2—C3	1.373 (7)	C9—H9C	0.9600
C2—C7	1.376 (6)	C10—O2	1.224 (5)
C3—C4	1.385 (7)	C10—C11	1.480 (6)
C3—H3	0.9300	C11—C12	1.382 (6)
C4—C5	1.383 (6)	C11—C16	1.398 (7)
C4—H4	0.9300	C12—C13	1.384 (6)
C5—C6	1.379 (6)	C12—H12	0.9300
C5—S	1.781 (5)	C13—C14	1.363 (8)
C6—C7	1.378 (6)	C13—H13	0.9300
C6—H6	0.9300	C14—C15	1.373 (6)
C7—H7	0.9300	C14—H14	0.9300
C8—O1	1.390 (4)	C15—C16	1.372 (6)
C8—C10	1.505 (5)	C15—H15	0.9300
C8—S	1.847 (4)	C16—H16	0.9300
			
C2—C1—H1A	109.5	O1—C9—H9A	109.5
C2—C1—H1B	109.5	O1—C9—H9B	109.5
H1A—C1—H1B	109.5	H9A—C9—H9B	109.5
C2—C1—H1C	109.5	O1—C9—H9C	109.5
H1A—C1—H1C	109.5	H9A—C9—H9C	109.5
H1B—C1—H1C	109.5	H9B—C9—H9C	109.5
C3—C2—C7	116.9 (5)	O2—C10—C11	120.0 (4)
C3—C2—C1	122.3 (5)	O2—C10—C8	119.2 (4)
C7—C2—C1	120.8 (5)	C11—C10—C8	120.7 (4)
C2—C3—C4	122.0 (5)	C12—C11—C16	117.9 (4)
C2—C3—H3	119.0	C12—C11—C10	118.1 (4)
C4—C3—H3	119.0	C16—C11—C10	123.9 (4)
C5—C4—C3	120.2 (5)	C11—C12—C13	120.6 (5)
C5—C4—H4	119.9	C11—C12—H12	119.7
C3—C4—H4	119.9	C13—C12—H12	119.7
C6—C5—C4	118.4 (5)	C14—C13—C12	120.9 (5)
C6—C5—S	121.0 (4)	C14—C13—H13	119.5
C4—C5—S	120.6 (4)	C12—C13—H13	119.5
C7—C6—C5	120.2 (5)	C13—C14—C15	119.0 (5)
C7—C6—H6	119.9	C13—C14—H14	120.5
C5—C6—H6	119.9	C15—C14—H14	120.5
C2—C7—C6	122.3 (5)	C16—C15—C14	121.0 (5)
C2—C7—H7	118.8	C16—C15—H15	119.5
C6—C7—H7	118.8	C14—C15—H15	119.5
O1—C8—C10	108.5 (4)	C15—C16—C11	120.4 (5)
O1—C8—S	113.6 (3)	C15—C16—H16	119.8
C10—C8—S	108.9 (3)	C11—C16—H16	119.8
O1—C8—H8	108.6	C8—O1—C9	113.7 (3)
C10—C8—H8	108.6	C5—S—C8	101.8 (2)
S—C8—H8	108.6		
			
C7—C2—C3—C4	−0.8 (7)	O2—C10—C11—C16	−177.7 (4)
C1—C2—C3—C4	178.2 (4)	C8—C10—C11—C16	2.3 (6)
C2—C3—C4—C5	1.0 (7)	C16—C11—C12—C13	1.2 (6)
C3—C4—C5—C6	−0.7 (6)	C10—C11—C12—C13	178.7 (4)
C3—C4—C5—S	177.5 (4)	C11—C12—C13—C14	0.5 (7)
C4—C5—C6—C7	0.3 (6)	C12—C13—C14—C15	−0.8 (7)
S—C5—C6—C7	−177.9 (4)	C13—C14—C15—C16	−0.6 (8)
C3—C2—C7—C6	0.3 (7)	C14—C15—C16—C11	2.4 (8)
C1—C2—C7—C6	−178.7 (4)	C12—C11—C16—C15	−2.7 (7)
C5—C6—C7—C2	0.0 (7)	C10—C11—C16—C15	180.0 (4)
O1—C8—C10—O2	−18.2 (5)	C10—C8—O1—C9	−164.3 (3)
S—C8—C10—O2	105.9 (4)	S—C8—O1—C9	74.4 (4)
O1—C8—C10—C11	161.7 (3)	C6—C5—S—C8	−83.1 (4)
S—C8—C10—C11	−74.1 (4)	C4—C5—S—C8	98.8 (4)
O2—C10—C11—C12	4.9 (6)	O1—C8—S—C5	63.2 (4)
C8—C10—C11—C12	−175.0 (4)	C10—C8—S—C5	−57.9 (3)

### Hydrogen-bond geometry (Å, º)

**Table:**

*D*—H···*A*	*D*—H	H···*A*	*D*···*A*	*D*—H···*A*
C1—H1*B*···O2^i^	0.96	2.49	3.366 (6)	152
C8—H8···O2^ii^	0.98	2.46	3.323 (6)	146

Symmetry codes: (i) −*x*+1, −*y*+2, *z*+1/2; (ii) −*x*+1, −*y*+2, *z*−1/2.
