# Supplementary figures and images for: Crystal structure of 2-meth­oxy-2-[(4-methyl­phen­yl)sulfan­yl]-1-phenyl­ethan-1-one
Source: Acta Crystallogr E Crystallogr Commun. 2015 Jan 1;71(Pt 1):o3–4. doi: 10.1107/S205698901402550X (PMC4331849; doi:10.1107/S205698901402550X)

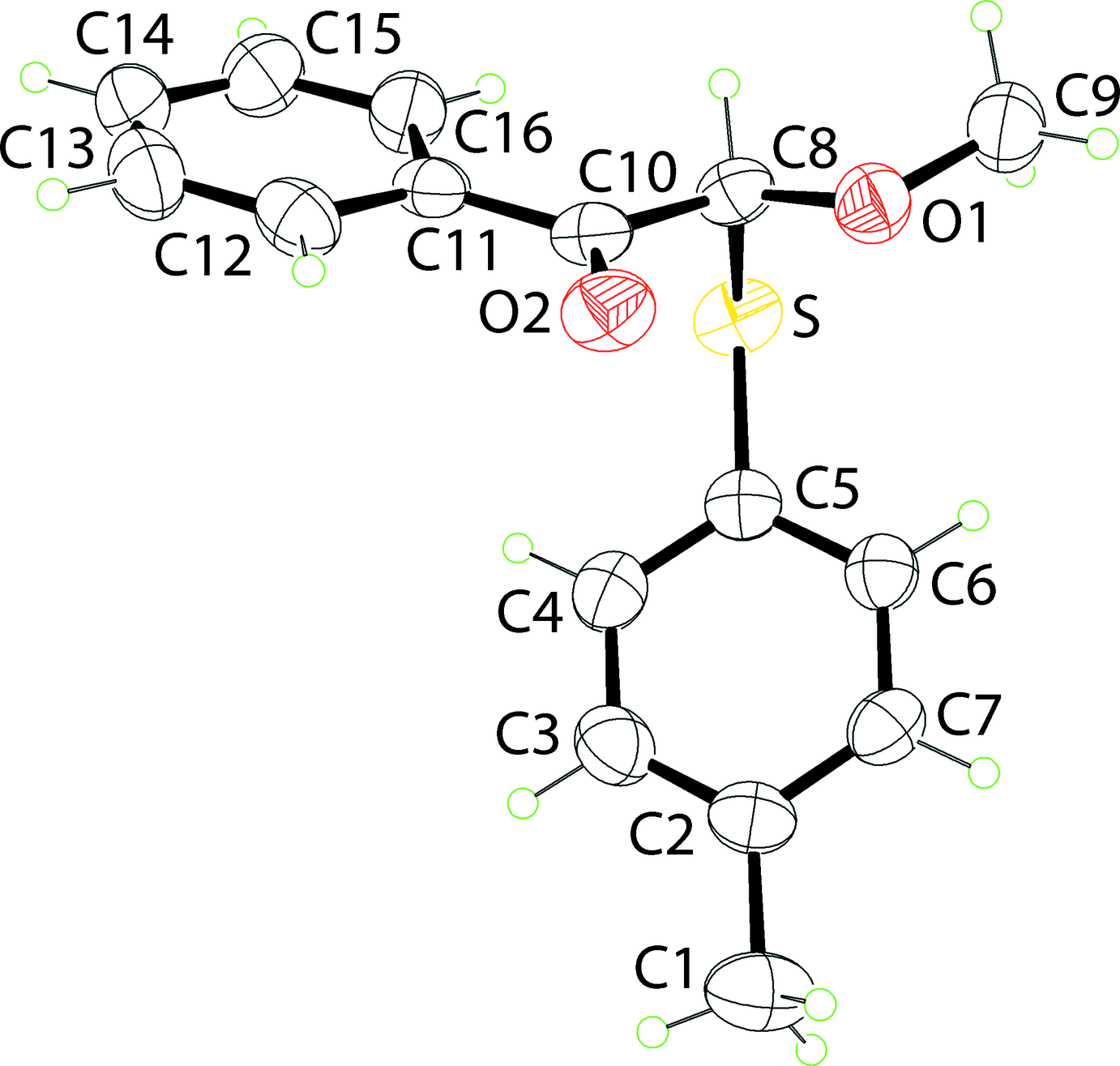

Supplement: Supplementary file 4 [file e-71-000o3-fig1.tif]

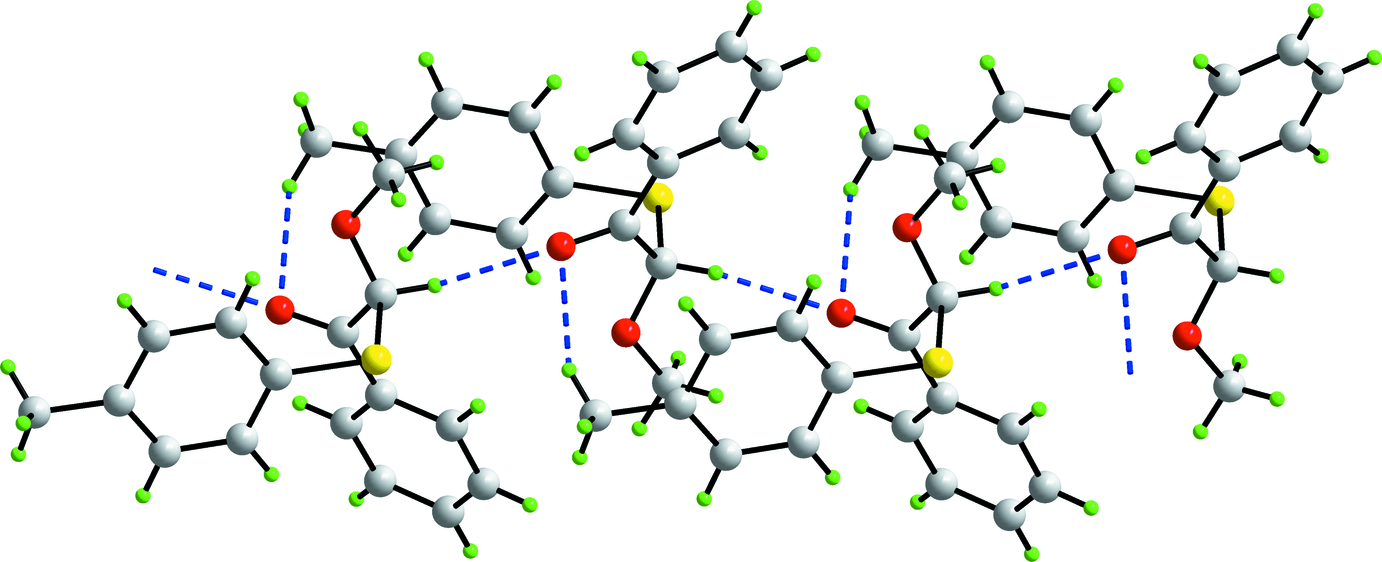

Supplement: Supplementary file 5 [file e-71-000o3-fig2.tif]

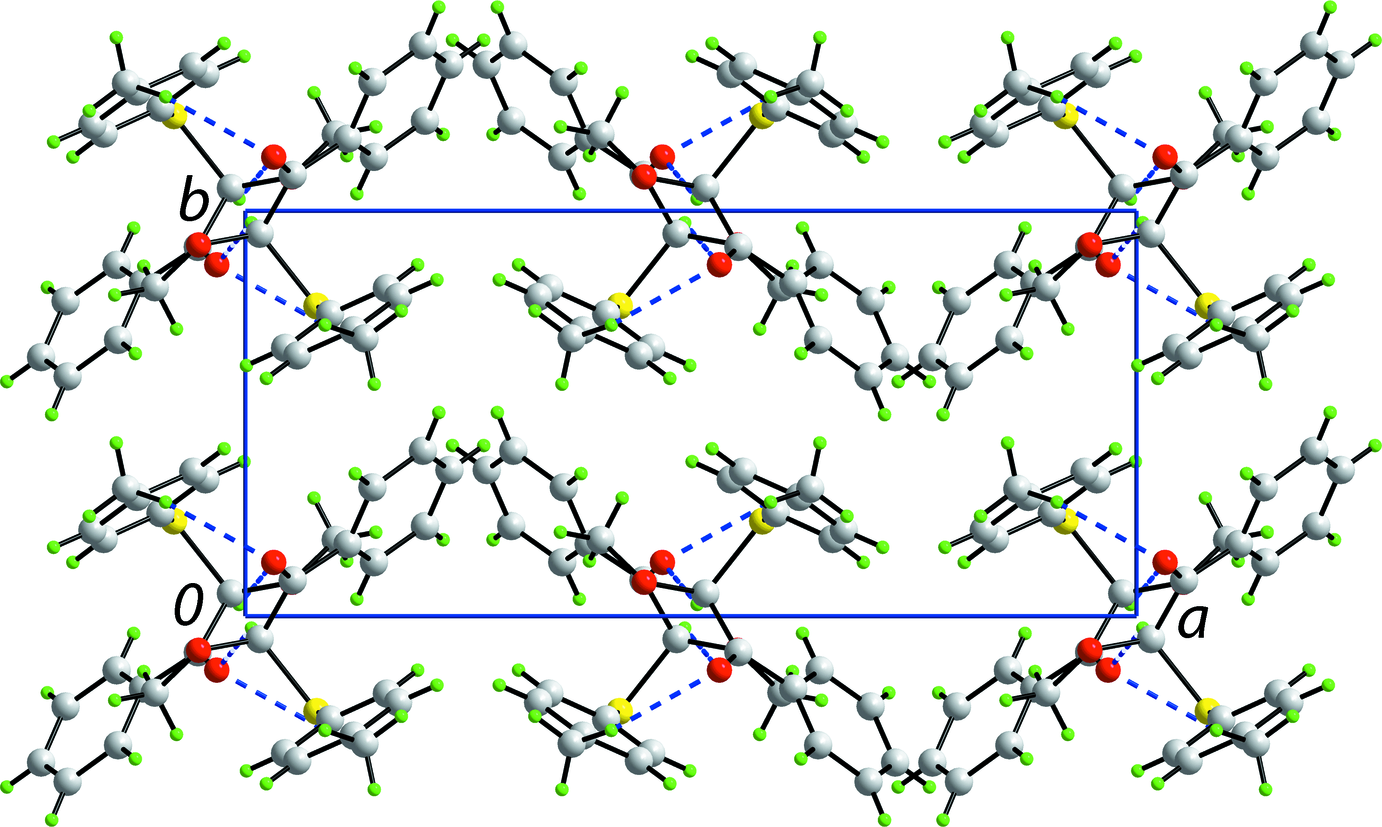

Supplement: Supplementary file 6 [file e-71-000o3-fig3.tif]
